# Metformin synergistically suppress tumor growth with doxorubicin and reverse drug resistance by inhibiting the expression and function of P-glycoprotein in MCF7/ADR cells and xenograft models

**DOI:** 10.18632/oncotarget.23187

**Published:** 2017-12-08

**Authors:** Ying Li, Meng Wang, Pei Zhi, Jian You, Jian-Qing Gao

**Affiliations:** ^1^ Institute of Pharmaceutics, College of Pharmaceutical Sciences, Zhejiang University, Hangzhou, P.R. China; ^2^ Zhejiang Province Key Laboratory of Anti-Cancer Drug Research, College of Pharmaceutical Sciences, Zhejiang University, Hangzhou, P.R. China

**Keywords:** metformin, doxorubicin, drug resistance, mitochondrial toxicity, P-glycoprotein

## Abstract

Acquired resistance to chemo-drugs remains a major obstacle to successful cancer therapy. Metformin, a well-documented drug for treating type II diabetes, was recently proposed as a novel agent for tumor treatment. In this study, we found that metformin suppressed MCF7/ADR, a doxorubicin-resistant breast cancer cell line, and acted synergistically with doxorubicin by reversing drug-resistant phenotypes both *in vitro* and *in vivo*. Metformin alone dose-dependently inhibited tumor growth, especially the stressful tumor microenvironment of glucose deficiency, and the cytotoxicity of metformin was markedly enhanced by increasing ROS production and ATP depletion. In addition, we found that metformin showed synergistic activity with doxorubicin against MCF7/ADR. Metformin increased nuclear doxorubicin accumulation and overcame drug resistance by down-regulating drug-resistant genes such as P-glycoprotein (Pgp). Metformin alone markedly inhibited MCF7/ADR tumor xenografts and demonstrated synergistic activity with doxorubicin *in vivo* by eliminating Ki67-positive cancer cells. In addition, metformin suppressed Pgp expression *in vivo*. In conclusion, our results suggested that metformin could potentially be used in the treatment of chemo-resistant tumors and could restore doxorubicin sensitivity.

## INTRODUCTION

Cancer recurrence after conventional chemotherapy remains a major challenge in clinical therapy of breast cancer due to increased multidrug resistance. Cancer cells acquire resistance to structurally and mechanistically unrelated chemotherapeutic drugs (e.g., doxorubicin, paclitaxel, docetaxel, and *Vinca* alkaloids) under selective pressure. Doxorubicin is a first-line chemo-drug. Although it effectively inhibits tumor growth, it otherwise enhances tumor malignancy, leading to cancer recurrence and poor responses to many conventional chemo-drugs. Thus, it is clinically imperative to identify agents for treating chemo-resistant tumors and restoring chemo-sensitivity.

Metformin is a well-established oral drug for type II diabetes that possesses high efficacy and safety. Epidemiological studies have shown that metformin use is associated with a lower incidence and mortality of numerous cancers, particularly in patients with type 2 diabetes [1, 2]. Accumulating pre-clinical evidence has shown the anti-tumor activity of metformin on different cancer cell lines, and registered clinical trials using metformin for cancer prevention or treatment have been approved [3, 4]. It has been found that metformin induces cell apoptosis and cell cycle arrest and inhibits tumor growth when used in *in vitro* or *in vivo* models [5]. In addition, metformin sensitizes tumor cells to traditional chemo-drugs as well as irradiation therapy [6–8]. Furthermore, metformin selectively eliminates the stem cell population from tumors, thereby preventing cancer recurrence [9, 10].

Recently, reports have indicated that metformin could overcome chemo-resistance in breast cancer, hepatocellular carcinoma and leukemia cells [11–13]. The overexpression of drug transporters is one of the most recognized mechanism of acquired drug resistance. For example, ATP binding cassette (ABC) family members mediate the drug efflux of various hydrophobic chemo-drugs and reduce the intracellular drug accumulation [14]. However, these pumps consume energy by hydrolyzing ATP to expel their substrates. It has been found that drug-resistant cancer cells exhibit higher ATP demand and are more susceptible to energy deprivation [15]. Hence, drugs targeting cancer metabolism, such as biguanides, could disrupt the energy supply and suppress the function of the drug pumps, which might facilitate the treatment of chemo-resistant tumors.

Currently, the detailed mechanisms for the anti-tumor activity of metformin remain elusive. Previous studies have indicated that metformin inhibits respiratory chain complex I, which is part of oxidative phosphorylation, and that it decreases the energy supply from mitochondria [16]. Depletion of ATP activates AMP-activated protein kinase (AMPK) and represses the mammalian target of rapamycin (mTOR), which limits the synthesis of biomolecules (such as protein or nuclear acid) and controls cell growth [17, 18]. In addition, metformin suppresses mitochondrial-dependent biosynthesis [19]. Further, metformin exerts indirect systematic effects by lowering the blood insulin or insulin-like growth factor-1 levels [20].

In the present study, we examined *in vitro* and *in vivo* anti-tumor effects in a doxorubicin resistant breast cancer cell line, i.e., MCF7/ADR. Of note, we emphasized the potential effect of tumor microenvironment (e.g., hypoxia or starvation) on the cytotoxicity of metformin. In addition, it was found that metformin reverses drug-resistant phenotypes by repressing the function and expression of drug transporters. Metformin acted synergistically with doxorubicin against MCF7/ADR in the cell line model or tumor xenografts. To our knowledge, our study provided *in vivo* studies of metformin on a doxorubicin-resistant breast tumor for the first time.

## RESULTS

### Metformin inhibited proliferation and induced apoptosis and cell cycle arrest in MCF7/ADR cells

**Figure 1:**
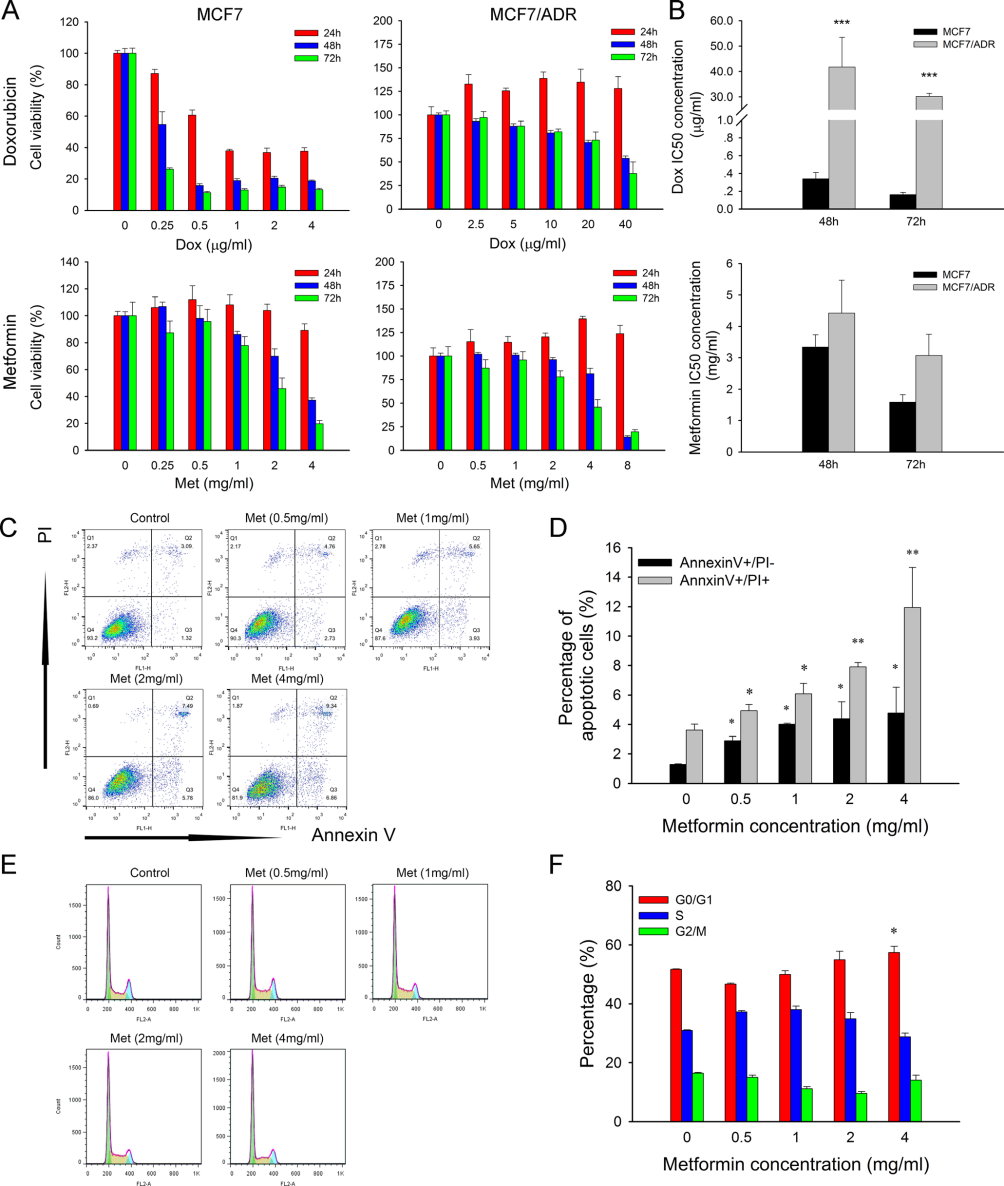
Cytotoxicity of metformin and doxorubicin (**A**) Effect of metformin and doxorubicin on cell proliferation of MCF7 and MCF7/ADR at 24, 48, 72 hours (*n* = 5). (**B**) IC50 concentration of doxorubicin and metformin. The data were obtained from three independent experiments. (**C**) Representative results of Annexin/PI staining after metformin treatment for 48 h in MCF7/ADR. (**D**) Statistical result showing the percentage of cells in early or late apoptosis (*n* = 3). (**E**) Representative results of cell cycle distribution after metformin treatment for 48 h in MCF7/ADR. (**F**) Statistical result showing the percentage of cells in G1, S or G2 phases (*n* = 3). Cells untreated were regarded as control (^*^P < 0.05, ^**^P < 0.01, ^***^P < 0.001).

To investigate the cytotoxicity of metformin, two breast cancer cell lines, i.e., MCF7 (doxorubicin-sensitive) and MCF7/ADR (doxorubicin-resistant), were used. MTT results showed that metformin caused dose and time dependent inhibition on both cell lines (Figure 1A). The IC50 of doxorubicin in MCF7/ADR cells was approximately 150 times over that of MCF7. However, the IC50 of metformin was only slightly higher in MCF7/ADR cells (Figure 1B), which indicated that MCF7/ADR did not show cross resistance to metformin.

Flow cytometry results showed that metformin promoted both early (Annexin V^+^/PI^-^) and late (Annexin V^+^/PI^+^) cell apoptosis in a dose-dependent manner (Figure 1C, 1D). However, metformin did not obviously alter the cell cycle distribution, but only the high level of 4 mg/ml induced G0/G1 arrest (Figure 1E, 1F).

### Metformin induced mitochondrial toxicity in MCF7/ADR

**Figure 2:**
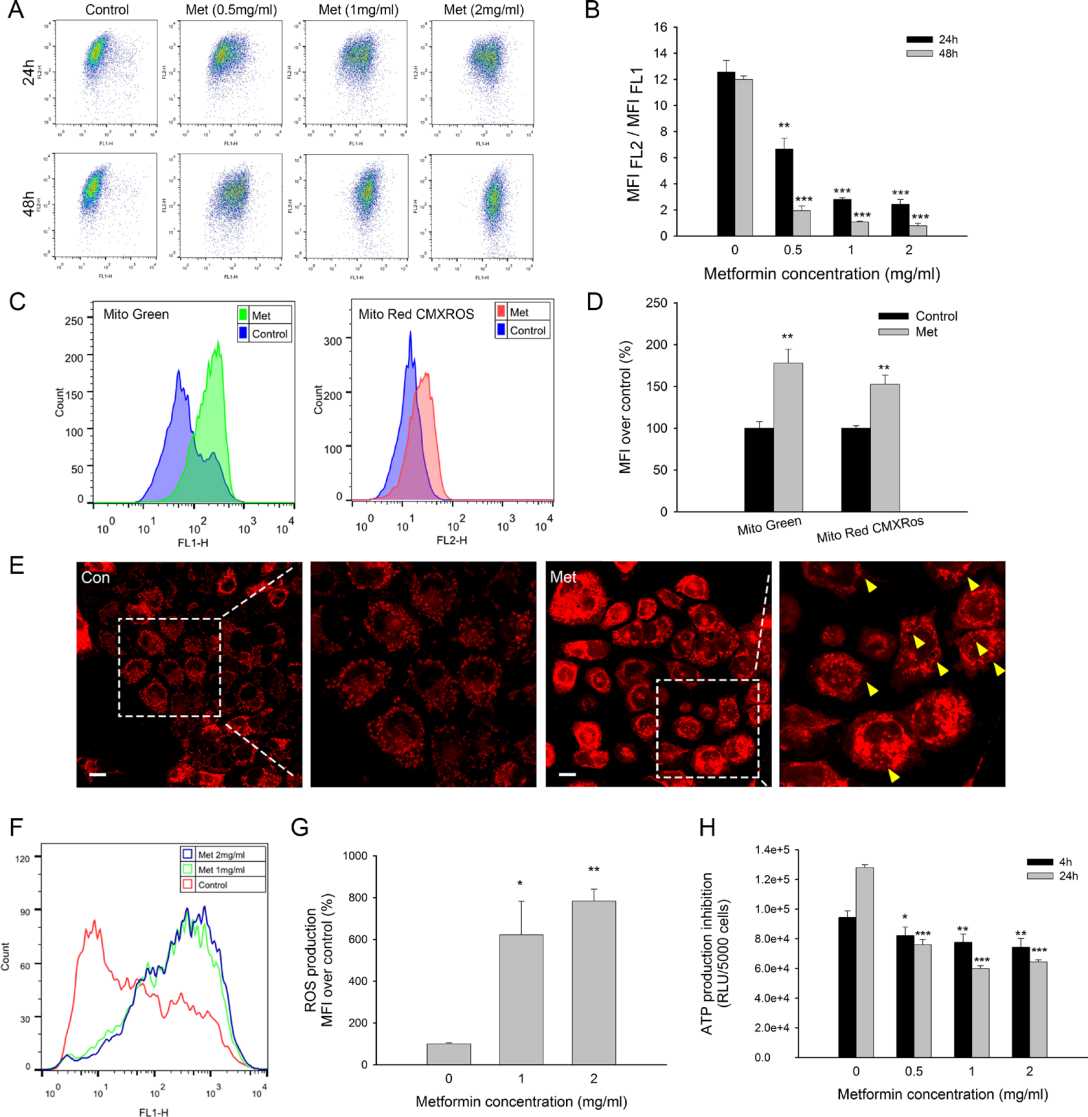
Mitochondrial toxicity of metformin in MCF7/ADR (**A**) Representative results of JC-1 staining after metformin treatment for 24, 48 h. (**B**) Statistical result of variation in mitochondrial membrane potential presented as MFI_(FL2)_ /MFI_(FL1)_ (Red/Green) (*n* = 3). (**C**) Representative flowcytometry results of mitochondrial mass staining using MitoTracker^®^ Green and MitoTracker^®^Red CMXRos after metformin treatment. (**D**) Statistical result of mitochondrial mass staining presented as MFI over control (*n* = 3). (**E**) CLSM results of mitochondrial mass staining after metformin treatment. Yellow arrows indicated the formation of vacuoles in the cytoplasm. Bar = 30 μm. (**F**) Representative results of DCFH-DA staining after drug treatment for 4 h. (**G**) Statistical result of ROS production (*n* = 3). (**H**) Inhibition of ATP production at 4, 24 h. The results were present as RLU (Relative light unit) per 5000 cells (*n* = 4). Cells untreated were regarded as control (^*^P < 0.05, ^**^P < 0.01, ^***^P < 0.001).

Metformin is considered an inhibitor of complex I of the electron transport chain in mitochondria [19]; therefore, mitochondrial toxicity is also considered one of the mechanisms of action by which metformin combats tumors. Metformin reduced the mitochondrial membrane potential in a time- and dose-dependent manner significantly, as shown by the decreasing ratio of MFI _(FL2)_/ MFI _(FL1)_ using JC-1 staining (Figure 2A, 2B). Low-dosage metformin treatment (0.5 mg/ml) at 24 h markedly induced the depolarization of mitochondria, which subsequently activated cell apoptosis. Interestingly, mito-probe staining showed that the mitochondrial mass was significantly increased after metformin treatment (Figure 2C). MitoTracker^®^ Green staining was used to evaluate the mitochondrial mass independent of the mitochondrial membrane potential, and MitoTracker^®^Red CMXRos fluorescence was dependent on both the mitochondrial mass and mitochondrial membrane potential [21]. Mitochondrial mass fluorescence was increased in both mito-probe staining, although MitoTracker^®^ Green showed relatively higher increases in signal than MitoTracker^®^Red CMXRos (Figure 2D). Weakened MitoTracker^®^Red CMXRos staining was probably due to a decreased mitochondrial membrane potential. Mitochondrial morphology was observed with MitoTracker^®^Red CMXRos. Metformin-treated cells showed blurred and aggregated staining, whereas the untreated cells displayed clear and dispersive mitochondrial morphology. Additionally, mounts of vacuoles could be observed in the cytoplasm of metformin-treated cells (Figure 2E).

Mitochondria are well documented as the main organelles of ROS formation and ATP production. To evaluate the mitochondrial function impaired by metformin, we further studied ROS and ATP production. Metformin treatment significantly promoted ROS production after 4 h of incubation (Figure 2F, 2G). The ATP levels were detected after 4 h and 24 h of metformin treatment to exclude the impact of dead cells because no significant cytotoxicity was observed in the early periods. Metformin substantially inhibited ATP production in a time-dependent manner, although concentrations over 1 mg/ml did not further decrease the ATP levels (Figure 2H). Taken together, these results showed that metformin exerts mitochondria toxicity by interrupting the normal function of mitochondria, including increasing the ROS levels and decreasing both the mitochondrial membrane potentials and the ATP levels.

### The tumor microenvironment affected the cytotoxicity of metformin

**Figure 3:**
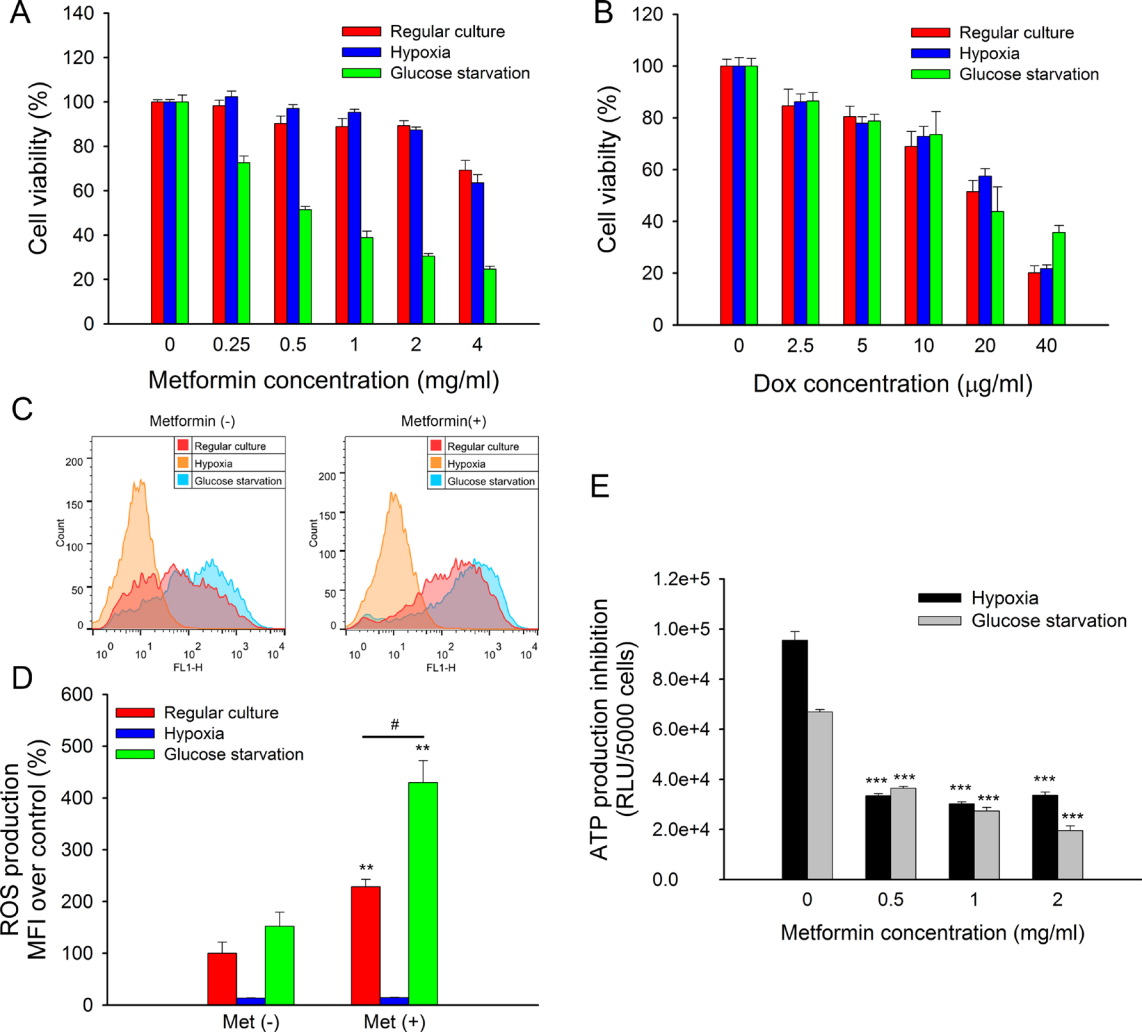
Impacted of hypoxia or glucose starvation on metformin in MCF7/ADR (**A**, **B**) Cytotoxicity of metformin (A) and doxorubicin (B) under regular culture, hypoxia or glucose starvation conditions for 48 h. The cell viability without drug treatment were designed as 100%, respectively (*n* = 5). (**C**) Representative results of DCFH-DA staining. ROS production with metformin treatment for 4 h under regular culture, hypoxia or glucose starvation. (**D**) Statistical result of ROS production (*n* = 3). (^*^P < 0.05, ^**^P < 0.01, compared with Met (–) groups; ^#^*P* < 0.05, compared with Met (+) group under regular culture) (**E**) ATP production of metformin treated MCF7/ADR under hypoxia or glucose starvation for 24 h. (*n* = 4). (^***^P < 0.001, compared with groups without metformin treatment).

The tumor microenvironment, which is characterized by hypoxia and nutrient deficiency, was generally different from regular cell culture conditions. It was found that hypoxia exhibited a higher risk of tumor progression and metastasis [22]. Moreover, the tumor microenvironment might impair the efficiency of anti-cancer drugs [23]. Here, we mimicked the tumor microenvironment with hypoxia and glucose starvation and assessed its influence on the cytotoxicity of metformin. The results showed that metformin cytotoxicity in MCF7/ADR was significantly enhanced by glucose starvation but not hypoxia (Figure 3A), whereas doxorubicin cytotoxicity was not affected (Figure 3B). Similarly, we confirmed that glucose deprivation also enhanced metformin toxicity in MCF7 and MDA-MB-231 but not doxorubicin (Supplementary Figure 1).

In addition, the mimicked tumor microenvironment could affect ROS and ATP production. ROS induced by metformin was further enhanced under glucose starvation, whereas ROS production was completely eliminated under hypoxia due to oxygen deprivation (Figure 3C, 3D). ATP depletion caused by metformin was unaffected by either hypoxia or glucose starvation after 24 h of treatment. However, total ATP production was significantly lower under glucose starvation than it was under hypoxia as well as under regular culture conditions (Figure 3E).

### Metformin exhibits *in vivo* anti-tumor effects in xenograft models

**Figure 4:**
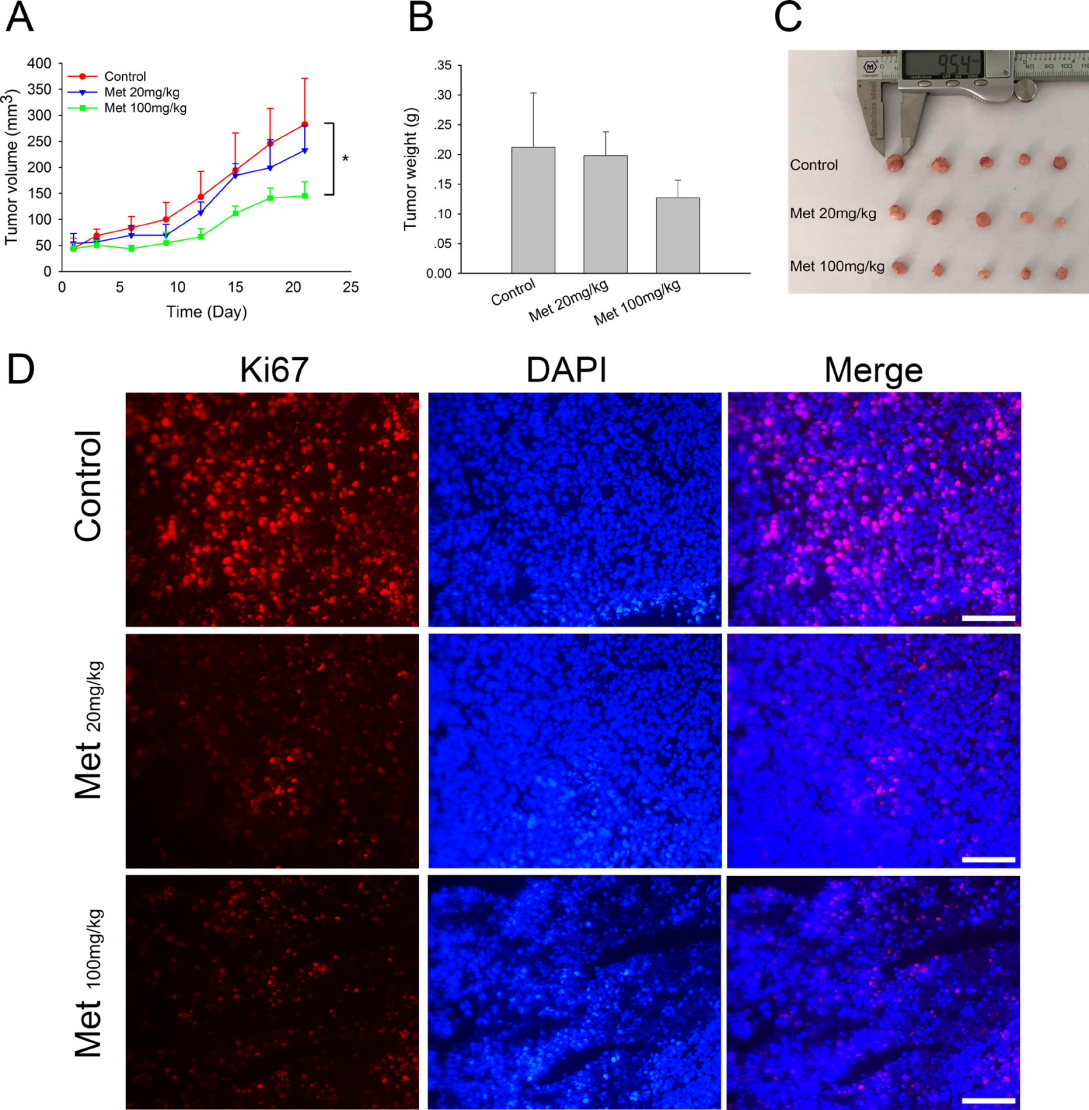
*In vivo* effect of metformin on MCF7/ADR xenografts (**A**) Tumor volume during metformin treatments. (**B**, **C**) The overall view and weight of the isolated MCF7/ADR tumors in the end of the treatments. Mice receiving intratumoral injection of physiological saline were regarded as control group. (^*^P < 0.05, compared with control group). (**D**) Immunofluorescence staining of Ki67 in MCF7/ADR tumor tissues. Bar = 100 μm.

To further study the *in vivo* anti-tumor effects of metformin in doxorubicin-resistant tumors, we established a xenograft model of MCF7/ADR by transplantation of fresh tumor tissue blocks. The intratumoral injection of metformin (100 mg/kg) significantly inhibited tumor growth after 21 days of treatment, exhibiting smaller tumor sizes and lower tumor weights. Lower doses of metformin (20 mg/kg) showed a relatively weaker inhibitory effect (Figure 4A–4C). These results suggested the direct anti-tumor effect of metformin on MCF7/ADR xenografts. In addition, Ki67 staining showed that metformin markedly eliminated actively dividing tumor cells *in vivo* (Figure 4D).

### The combined effect of doxorubicin and metformin in MCF7/ADR

**Figure 5:**
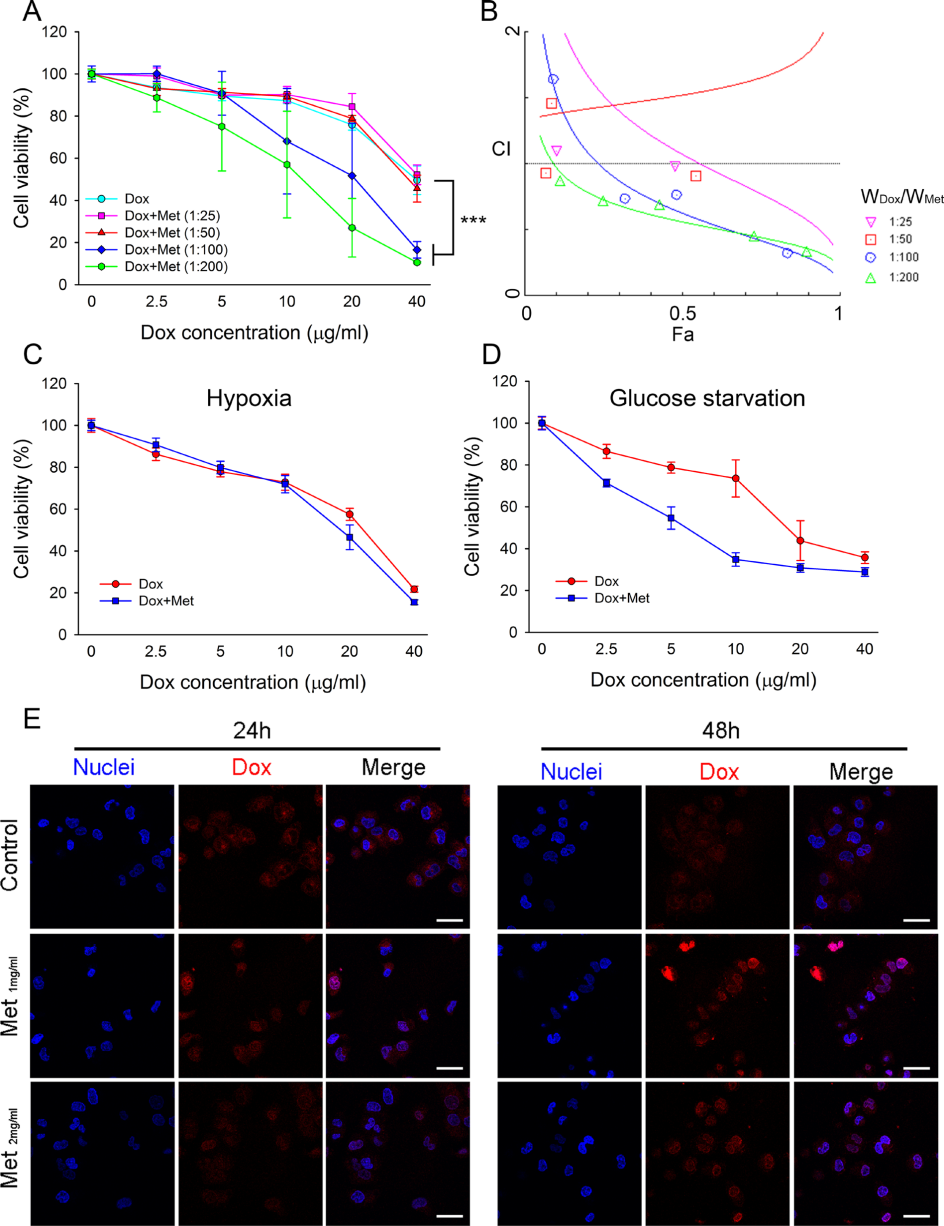
Combined effect of metformin with doxorubicin in MCF7/ADR (**A**) Effect of metformin and doxorubicin with defined combination ratios on cell proliferation at 48 h (*n* = 5), (^***^P < 0.001, compared with Dox group). (**B**) The Fa-CI plot shows the combination index (CI) value for different ratio of metformin and doxorubicin using CalcuSyn software. Fa represents effect levels. (**C**, **D**) The combination effect of doxorubicin and metformin under hypoxia (C) or glucose starvation (D) for 48 h with fixed ratio of Dox:Met (1:100; w/w). The cell viability without drug treatment were designed as 100%, respectively (*n* = 5) (**E**) Doxorubicin distribution in MCF7/ADR with metformin pre-treatment. Cell nuclei were stained with Hochest33342. Bar = 30 μm.

As MCF7/ADR possessed high resistance to doxorubicin, we next investigated whether metformin could re-sensitize MCF7/ADR to doxorubicin and reverse drug resistance. The combination of doxorubicin and metformin against MCF7/ADR was assessed with fixed ratios. Doxorubicin-metformin co-incubation with MCF7/ADR significantly enhanced cytotoxicity compared with a single drug (Figure 5A). The CI value was calculated using the Chou–Talalay method [24], as shown in the Fa-CI plot (synergism, CI < 1; additivity, CI = 1; antagonism, CI > 1) (Figure 5B; Table 1). It was found that a high ratio of W_Met_/W_Dox_ showed synergistic anti-proliferation in MCF7/ADR. However, the combination did not show synergistic inhibition on doxorubicin-sensitive cell lines (MCF7 or MDA-MB-231), probably due to the overwhelming cytotoxicity of doxorubicin (Supplementary Figure 2). The combined effect of doxorubicin and metformin under hypoxia or glucose starvation was also studied. With a fixed ratio (W_Dox_/W_Met_=1:100), the synergistic effect was reduced under hypoxia, but metformin still significantly increased the cytoxicity of doxorubicin under glucose starvation (Figure 5C, 5D).

**Table 1:** Combination effects of doxorubicin and metformin against MCF7/ADR for 48 h

Dox:Met (W/W)	CI			R^2^
	ED50	ED75	ED90	
1:25	1.07283	0.77190	0.56712	0.92922
1:50	1.52276	1.65528	1.84752	0.92512
1:100	0.62623	0.41786	0.28635	0.98882
1:200	0.56038	0.44170	0.35569	0.99688

Doxorubicin accumulation was reduced in the cytoplasm and nuclei of MCF7/ADR, which was closely associated with drug resistance due to the overexpression of drug transporters. We next studied whether metformin enhanced the cellular uptake of doxorubicin. MCF7/ADR was pre-treated with metformin for 24 h or 48 h before doxorubicin incubation. The confocal laser scanning microscope (CLSM) results demonstrated that doxorubicin accumulation in MCF7/ADR was enhanced by metformin pretreatment. Moreover, a stronger nuclear doxorubicin distribution was observed in the metformin pre-treated MCF7/ADR (Figure 5E). This might explain the synergistic effect of metformin by increasing intracellular doxorubicin accumulation.

### Metformin reversed chemo-induced drug resistance

**Figure 6:**
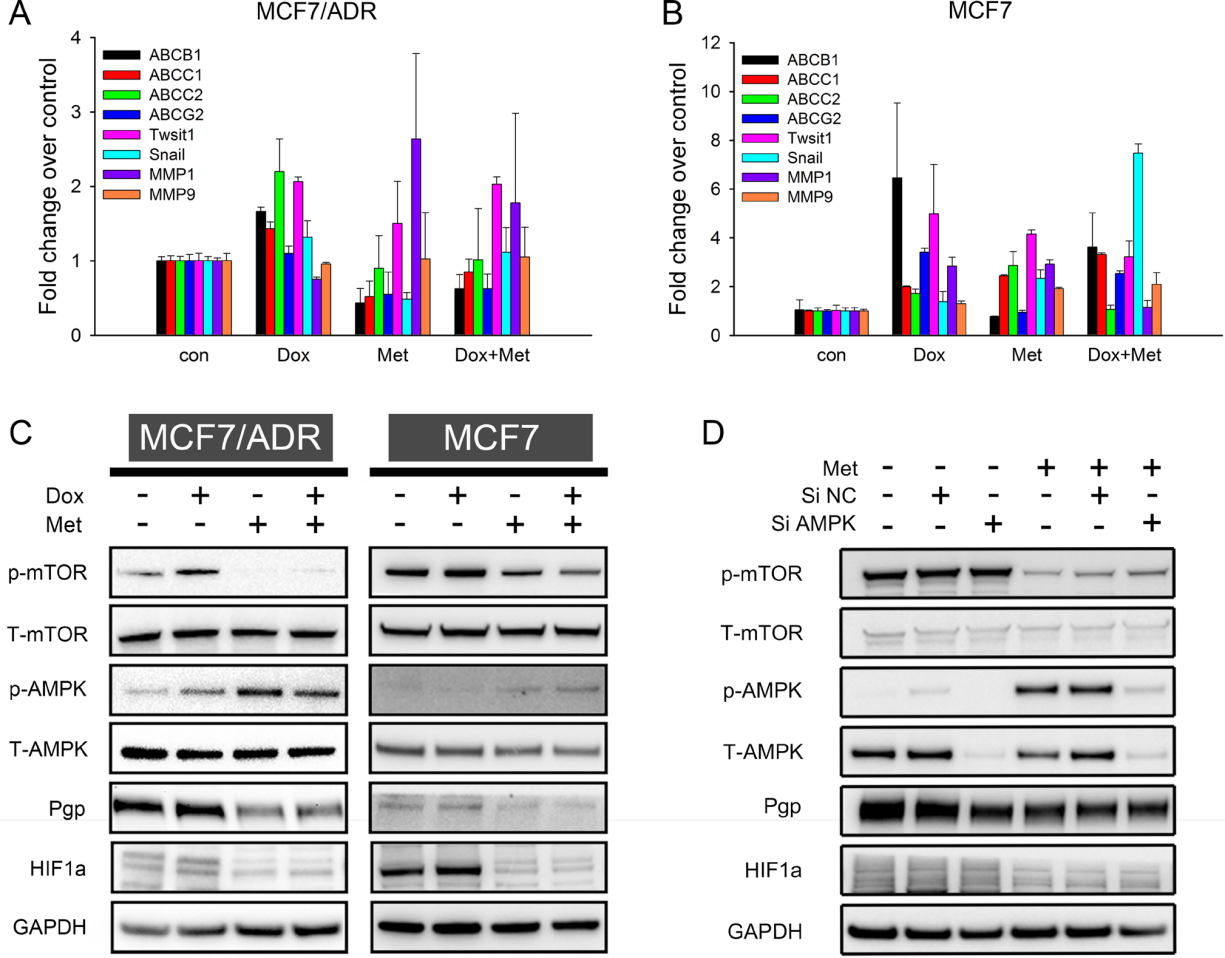
Mechanism studies of doxorubicin-metformin combined treatment (**A**, **B**) qRT-PCR results showed fold change of gene expression in MCF7/ADR (A) and MCF7 (B) after 48 h treatment (*n* = 3). Cells untreated were regarded as control. (**C**) Representative western blot results after 48 h treatment in MCF7 and MCF7/ADR. (**D**) Representative western blot results of metformin in AMPK knockdown MCF7/ADR. GAPDH was included as internal control.

Prolonged chemotherapy induced drug resistance is usually characterized by tumor cells overexpressing various ATP-binding cassette transporters and exhibiting more invasive phenotypes than their parental cells [25]. To elucidate the synergism of doxorubicin and metformin and explore the possibility of metformin overcoming drug resistance, we measured the expression of the relevant genes. The qRT-PCR results (Figure 6A) showed that doxorubicin up-regulated the drug resistant genes (*ABCB1, ABCC1, ABCC2,* and *ABCG2*) in MCF7/ADR, whereas metformin significantly down-regulated them. For doxorubicin-metformin combined treatment, the expressions of drug resistance genes were repressed compared with single-doxorubicin treatment. The epithelial mesenchymal transition (EMT) has been reported to be closely associated with increasing cancer aggressiveness, including drug resistance and tumor metastasis [26]. Here, several tumor EMT markers (*Twist1, Snail, MMP1,* and *MMP9*) were evaluated, but metformin did not inhibit EMT in MCF7/ADR. Furthermore, we assessed the impact of metformin on chemo-induced drug resistance in MCF7. This drug sensitive cell line might have acquired resistance via doxorubicin stimulation [27]. Similarly, doxorubicin robustly enhanced the expression of several drug resistance genes, but metformin alone or in combination reversed them (especially *ABCB1* and *ABCG2*) in MCF7 (Figure 6B). Taken together, these results indicated that whereas doxorubicin significantly enhanced a drug resistance phenotype, metformin could reverse this transition.

The AMPK/mTOR pathway is one of the most recognized anti-tumor mechanisms of metformin, so we next investigated whether AMPK/mTOR activation is involved in overcoming drug resistance. As expected, increasing AMPK phosphorylation and decreasing mTOR phosphorylation were observed with metformin alone or in combined treatments, thus suggesting the activation of AMPK/mTOR in MCF7/ADR. Similar results were also observed in MCF7 (Figure 6C). Pgp (coded by *ABCB1*) expression was confirmed on a protein level, consistent with the qRT-PCR results. Metformin alone repressed Pgp expression in both cell lines, whereas doxorubicin stimulated Pgp expression. In the combined treatment, metformin also reduced Pgp expression compared with doxorubicin alone, indicating that metformin could reverse doxorubicin-induced drug resistance. HIF1a is a well-known transcription factor that promotes drug resistance by enhancing Pgp expression, especially under hypoxia [28–30]. Recently, HIF1a was identified as a downstream target regulated by mTOR [31]. Hence, we examined HIF1a expression on a protein level. Metformin alone or in combination significantly repressed HIF1a. These results indicated that metformin activated the AMPK/mTOR axis in MCF7/ADR and MCF7 cell lines and further reversed the drug resistant phenotype with decreased Pgp and HIF1a expressions.

Since AMPK is an important target of metformin, we next investigated the effect of metformin in AMPK knockdown MCF7/ADR cells. Transfection of siAMPK significantly reduced both total and phosphorylated AMPK level at 48 h. Metformin was then added for another 48 h. The results showed that the metformin-induced AMPK activation was remarkably blocked but that mTOR repression was only slightly reversed (Figure 6D). Previous studies reported mTOR to be a downstream molecule regulated by AMPK [32]. However, our results indicated that metformin could directly inhibit mTOR in an AMPK independent way. Additionally, the Pgp and HIF1a protein levels were not significantly impacted by AMPK knockdown (Figure 6D).

### Metformin enhanced the anti-tumor effects of doxorubicin in xenograft models

**Figure 7:**
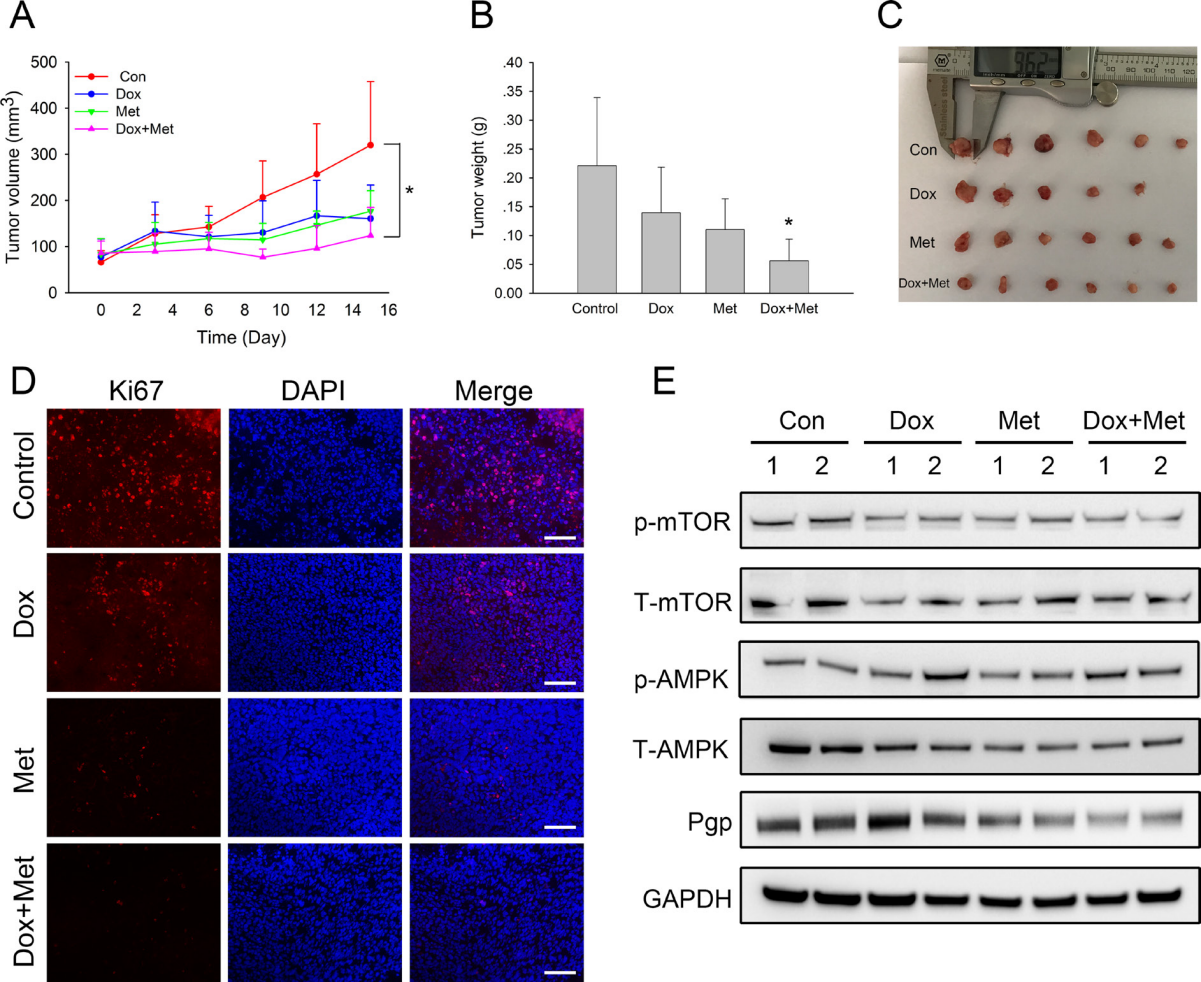
*In vivo* combined effect of metformin and doxorubicin on MCF7/ADR xenografts (**A**) Tumor volume during drug treatments. (**B**, **C**) The overall view and weight of the isolated MCF7/ADR tumors in the end of the treatments. (^*^P < 0.05, compared with control group). (**D**) Immunofluorescence staining of Ki67 in MCF7/ADR tumor tissues. Bar = 100 μm. (**E**) Western blot results of isolated MCF7/ADR tumors after drug treatments.

We next examined whether metformin had synergistic anti-tumor effects with doxorubicin in the xenograft model of MCF7/ADR. The results showed that both metformin and doxorubicin alone inhibited tumor growth with smaller tumor volumes and lower tumor weights at the end of the treatment. Notably, the combination of metformin and doxorubicin had the most significant inhibitory effect (Figure 7A–7C). Moreover, immunofluorescence results also indicated that fewer Ki67 positive cells existed in drug treated tumor tissues, within which the combination treatment had the least Ki67 staining (Figure 7D). Alexa Fluor^®^488 labeled secondary antibodies were used as well, which showed that doxorubicin did not interfere with the immunofluorescence staining results (Supplementary Figure 3). Taken together, these results showed that metformin enhanced the effect of doxorubicin against drug resistant tumors *in vivo*. We then examined whether metformin could reverse drug-resistant phenotypes in the MCF7/ADR xenograft model. The western blot results showed that metformin alone or combined markedly reduced Pgp expression in the tumor tissue but that doxorubicin did not. Additionally, mTOR repression was observed in drug treated tumor tissues (Figure 7E). These results indicated that in addition to the synergistic anti-tumor effects with doxorubicin, metformin reversed drug resistance by repressing Pgp expression in xenograft models.

## DISCUSSION

An increasing number of studies has indicated that metformin has potential anti-tumor activity in numerous cancers, although the underlying mechanisms are still elusive. The prolonged use of doxorubicin in chemotherapy leads to the development of multidrug resistance, which appears to be a severe obstacle for cancer therapy. Recently, it was found that metformin could reverse chemo-resistance in various tumor cell lines [11–13, 33, 34]. In this study, we focused on the *in vitro* and *in vivo* effects of metformin against doxorubicin-resistant tumors using a breast cancer cell line, i.e., MCF7/ADR, and its synergistic effects with doxorubicin on reversing chemodrug-induced resistance.

The MCF7/ADR cell line, which possesses high resistance to doxorubicin due to overexpression of drug-efflux ‘pump’ such as Pgp, does not show cross-drug resistance to metformin. Previous reports showed that metformin is not the substrate for Pgp, which avoids Pgp mediated drug efflux [11]. As in our study, metformin exhibited similar cytotoxicity in MCF7 and MCF7/ADR. Therefore, metformin could be used in the treatment of doxorubicin-resistant tumors.

Mitochondria are the energy factories of the cells, and they supply ATP via oxidative phosphorylation [19]. Thus, complex I inhibition by metformin enhanced the generation of ROS and decreased ATP production [35]. Mitochondrial dysfunction caused by metformin suggested mitochondria as a target in MCF7/ADR. A recent study reported that metformin with mitochondria-targeting modification further enhanced cytotoxicity in pancreatic cancer cells, which emphasized the role of mitochondria in the anti-tumor mechanisms of metformin [35]. Interestingly, we found increasing mitochondrial masses after metformin treatment. A previous study suggested that cells increase the mitochondrial mass in response to oxidative stress [21, 36]. This phenomenon might be explained as metformin-stimulated ROS production. Additionally, an increased mitochondrial mass might be cellular self-protection to compensate for a low energy supply. Although metformin induced mitochondrial toxicity in various tumor cells, there were several studies reported the protective effect of metformin against oxidative damage in cardiomyocytes or neuron cells [37, 38]. This might be associated with intracellular ROS balance. Tumor cells possess higher level of ROS than normal cells and are more dependent on the antioxidant defense system, which might make them more vulnerable to ROS damage [39].

Increasing evidence has suggested that cells residing in solid tumors are under a stress microenvironment due to lack of angiogenesis [40]. It is suggested that the tumor microenvironment could affect the efficacy of anticancer drugs [23]. In this regard, we further investigated the anti-tumor effect of metformin under hypoxia or glucose starvation. Glucose starvation significantly enhanced the cytotoxicity of metformin but not hypoxia, whereas hypoxia and glucose starvation did not significantly affect the cytotoxicity of doxorubicin. Recently, other studies also reported sufficient nutrient supply inhibited the cytotoxicity of metformin [41, 42]. Metformin can induce a metabolic shift from mitochondrial respiration to glycolysis [43, 44]. As glycolysis generates energy inefficiently, the cancer cells consume more glucose. Therefore, metformin treatment under glucose starvation enhanced the “energy crisis” in tumor cells. In addition, ROS-induced DNA damage, together with decreasing ATP levels, could inhibit DNA repair and ultimately lead to cell death. Hypoxia is usually found to co-occur with low glucose within tumor sites [23]. Oxygen deprivation repressed oxidative phosphorylation and forced tumor cells to switch to aerobic glycolysis [45]. Increased glycolysis generating less ATP, which explained the ATP inhibition of metformin was not affected under hypoxia. ATP is the intracellular “energy currency”, which accounts for many important biological functions, such as biosynthesis and DNA damage repair. Notably, for drug-resistant cells, ATP is also the provider of energy for the drug ‘pumps’. Taken together, the tumor microenvironment could impact the toxicity of metformin by interrupting the cellular metabolic balance. Glucose starvation with metformin-induced massive ATP depletion was especially lethal to tumor cells. It was recently found that the cytotoxicity of metformin is affected by medium-renew or cell density, which might be associated with glucose exhaustion in the culture medium [41].

Multidrug resistance appears to be one of the most challenging problems in chemotherapy. For example, doxorubicin induces drug resistance by overexpressing numerous drug transporters (e.g., *ABCB1*, *ABCC1*, *ABCC2*, and *ABCG2*) to reduce intracellular drug accumulation. *ABCB1* encoded Pgp is up-regulated in various drug resistant tumors and accounts for the efflux of different drugs (MCF7/ADR used in our study showed cross-resistance to paxitacel). The inhibition of Pgp expression or function is feasible to reverse multidrug resistance. Our results showed that the combination of doxorubicin and metformin exhibited synergistic effects in MCF7/ADR. Since Pgp consumes two ATPs to export one substrate [14, 33], metformin could impair Pgp function by reducing ATP production. This leads to increased intracellular and nuclear doxorubicin accumulation, which facilitates doxorubicin cytotoxicity. Furthermore, metformin inhibited *ABCB1* (Pgp) expression on the mRNA and protein levels, whereas doxorubicin enhanced the drug-resistant phenotype. This result suggested that metformin might reverse acquired multidrug resistance during prolonged doxorubicin treatment. Additionally, it was found that metformin could selectively eliminate cancer stem cells, which are responsible for intrinsic drug resistance [46, 47] and prolong remission in multiple cancer cell types [9, 10]. Taken together, metformin could resensitize MCF7/ADR to doxorubicin and reversed drug resistance, suggesting its potential application in overcoming drug resistance in chemotherapy.

The AMPK/mTOR axis is a well-documented cell signal pathway that is frequently used to elucidate the anti-tumor mechanism of metformin. AMPK is a key energetic sensor and is activated by a decreasing intracellular ratio of ATP/AMP, whereas activated AMPK suppresses the mTOR pathway via tuberous sclerosis 2 protein (TSC-2) [32, 48]. Activated mTOR is closely associated with tumor genesis by promoting cell proliferation and the biogenesis of macromoleculars [49]. Recently, it was demonstrated that targeting AMPK or the mTOR signaling pathway could overcome drug resistance [50–52]. Metformin activated AMPK and inhibited mTOR in both MCF7 and MCF7/ADR. HIF1a is an important transcription factor that helps the tumor cells acquire aggressive and drug-resistant phenotypes [28], and it is identified as a down-stream molecule of the mTOR signal pathway [31]. Herein, our study found that HIF1a repression was consistent with mTOR inhibition, which might also explain decreased Pgp expression. Similar studies reported metformin inhibited HIF further suppress tumor angiogenesis by reducing VEGF levels [53].

Although many studies have suggested that the AMPK/mTOR axis has a key role in anti-tumor mechanisms of metformin, our results showed that mTOR suppression by metformin is only partly dependent on AMPK activation. Other studies have reported that metformin inhibited mTOR activity in an AMPK-independent way [54, 55]. REDD1 has been identified as a negative regulator of mTOR and a new molecular target of metformin in prostate cancer cells [54]. Pgp or HIF1a repression was also not affected by AMPK knockdown. These results indicated that metformin exhibits an AMPK-independent mechanism against MCF7/ADR. However, studies have described AMPK-dependent drug resistance or EMT reversal by metformin [13, 34], possibly due to the different cell models or culture conditions used.

Currently, *in vivo* anti-tumor studies of metformin reversing drug-resistant are still limited. Previous studies found metformin could overcome drug resistance to trastuzumab or gefitinib in xenograft tumor models [46, 56]. To our knowledge, this is the first *in vivo* study on metformin in a doxorubicin-resistant breast tumor xenograft model. Our results suggested that metformin alone could inhibit the tumor growth of MCF7/ADR xenografts and further enhance doxorubicin cytotoxicity in the combined treatment. Importantly, metformin reversed the multidrug resistant phenotype *in vivo*. Although doxorubicin efficiently suppresses most tumor growth, it might lead to multidrug resistance [25, 27]. Co-delivering metformin with doxorubicin reduced Pgp expression, which might prevent multidrug resistance development and resensitize the chemo-resistant tumors. Recent studies found that it is more effective to treat sarcoma by using liposomes coencapsulated with metformin and epirubicin, which eliminate cancer stem cells and prevent tumor recurrence [57].

However, the current studies all require high doses of metformin, which might cause side effects and limit its clinical application. Metformin is a hydrophilic drug whose cellular uptake is dependent on organic cation transporters (e.g., OCT1). Therefore, the cancer heterogeneity of OCT1 expression could affect metformin sensitivity [58]. In this regard, modifying metformin itself or developing drug carriers might further improve the therapeutic efficiency [57, 59].

**Figure 8:**
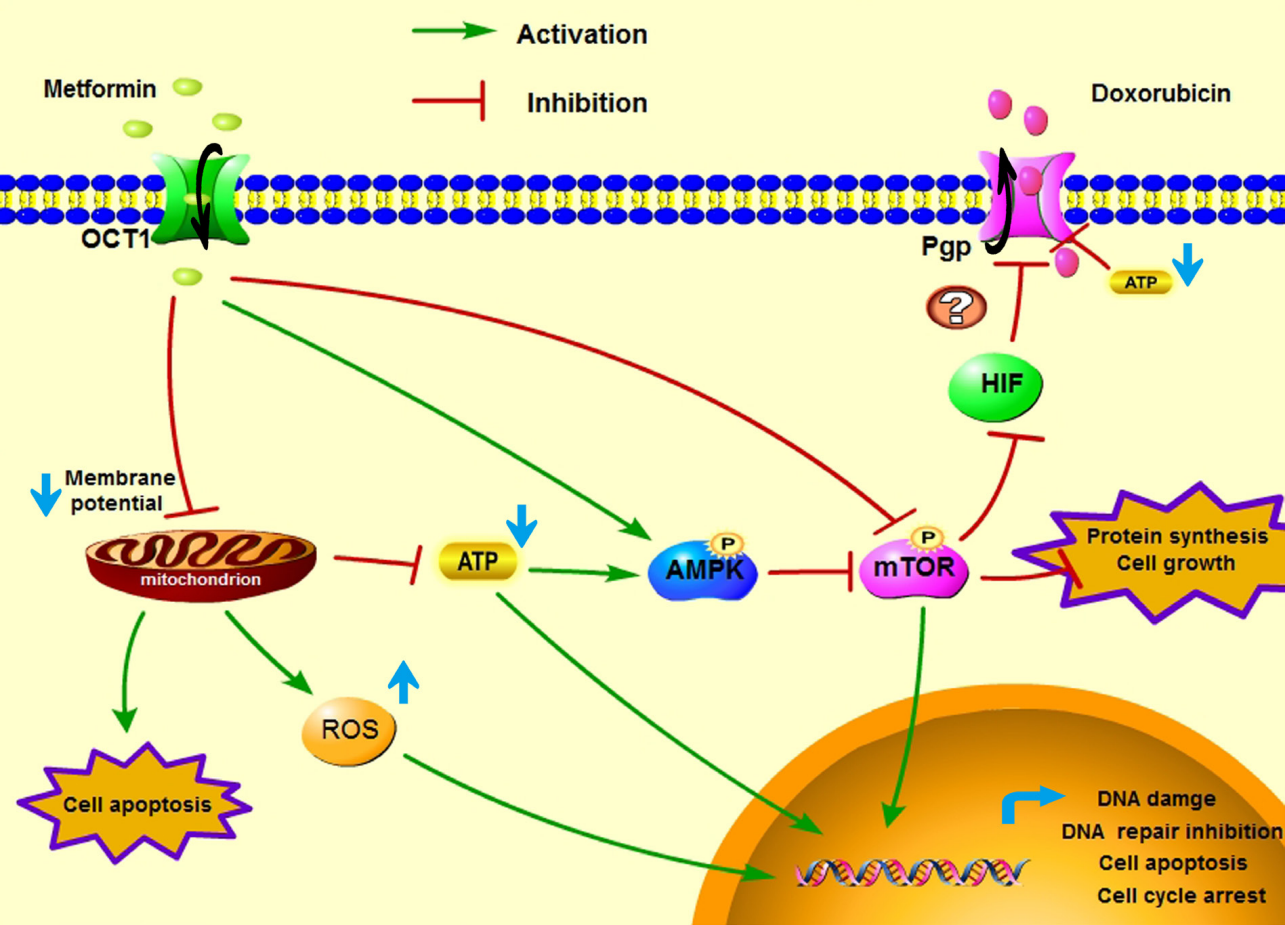
A proposed graphical mechanism illustrates the possible anti-tumor effect of metformin in MCF7/ADR Metformin induces mitochondrial toxicity and activates the AMPK/mTOR signal pathway, resulting cell apoptosis, DNA damage and protein synthesis inhibition, which suppressed the tumor cell growth. Additionally, metformin inhibits Pgp expression and depletes ATP production, which increases intracellular doxorubicin accumulation and displays synergistic effects with doxorubicin against MCF7/ADR cells.

In conclusion, our study demonstrated metformin showed *in vivo* and *in vitro* anti-tumor activity on the doxorubicin-resistant breast cancer cells and overcame drug resistance by inhibiting Pgp function and expression. We emphasized the impact of the tumor microenvironment on metformin toxicity in MCF7/ADR cells and found that glucose starvation significantly enhanced the cytotoxicity of metformin. In addition, metformin enhanced the anti-tumor effects of doxorubicin and reversed drug resistance by inhibiting Pgp both *in vitro* and *in vivo*. The proposed mechanism of metformin exerting anti-tumor activity against MCF7/ADR and exerting synergistic effects with doxorubicin is shown in Figure 8. Taken together, metformin alone or combined with doxorubicin is promising in treating chemo-resistant tumors.

## MATERIALS AND METHODS

### Reagents

Metformin (Met) was purchased from Dalian Meilun Biotech Co. Ltd. (Dalian, China). Doxorubicin (Dox) was obtained from Zhejiang Hisun Pharmaceutical Co. Ltd. (Taizhou, China). 3-(4,5-dimethylthi-zaol-2-yl)-2,5-diphenyltetrazolum bromide (MTT) was purchased from Sigma-Aldrich (Shanghai, China). The cell culture medium and trypsin were all purchased from Jinuo Biomedical Technology (Hanghzou, China). Fetal bovine serum (FBS) was purchased from Sijiqing Biologic Co., Ltd. (Hangzhou, China). An annexin V-FITC/PI assay kit, cell cycle detection kit and BCA Protein assay kit were bought from Nanjin KeyGEN Biotech Co. Ltd. (Nanjin, China). A Reactive Oxygen Species Assay Kit, Mitochondrial Membrane Potential Assay Kit with JC-1, RIPA lysis buffer and BeyoECL Plus Kit were purchased from the Beyotime Institute of Biotechnology (Jiangsu, China). Primary antibodies against phospho-mTOR (p-mTOR), total mTOR (T-mTOR), total AMPK (T-AMPK), Pgp, Ki67 were obtained from the Abcam Company (Cambridge, UK). The anti-phospho-AMPK (p-AMPK) antibody was obtained from Cell Signal Technology (Beverly, USA). The anti-HIF1a antibody was from Proteintech group (Chicago, USA). The anti-GAPDH antibody was bought from Goodhere Biotechnology (Hangzhou, China). Secondary antibodies HRP or Alexa Fluor^®^594 conjugated goat anti-rabbit IgG, MitoTracker^®^ Green FM, MitoTracker^®^ Red CMXRos, Hochest33342 and NuPAGE^®^ Bis-Tris Pre-Cast Gels were purchased from Life Technology (NY, USA). RNAiso Plus and PrimeScript™ RT Master Mix were purchased from Takara (Nojihigashi, Japan). GoTaq® qPCR Master Mix and Celltiter-Glo^®^ Luminescent Kit was bought from Promega (WI, USA). A RFect Transfection Reagent was purchased from Biodai Biotechnology (Changzhou, China).

### Cell culture

The doxorubicin resistant cell line MCF-7/ADR cell line was kindly provided by the Cancer Research Center of the Second Affiliated Hospital of Zhejiang University School of Medicine, and maintained in RPMI-1640 medium (containing 2 mg/ml D-Glucose) supplemented with 10% FBS, 100 U/ml penicillin and 100 μg/ml streptomycin. Doxorubicin (1 μg/ml) was added to the medium once every 1–2 weeks to maintain drug resistance. The cells were cultured in incubators maintained at 37°C with 5% CO_2_ under fully humidified conditions (referred as a regular culture below).

Hypoxia was achieved using a hypoxia incubator (Thermo Fisher) with a mixture of gas (94% N_2_, 5% CO_2_ and 1% O_2_) at 37°C. Glucose starvation was conducted by replacing the regular culture medium with D-Glucose free RPMI-1640.

### Cell viability

Cells were inoculated into 96-well plates at a density of 5 × 10^3^/well. Drugs of different concentrations were added the next day and incubated for determined periods. MTT solution was added at a final concentration of 0.5 mg/ml and incubated at 37°C for another 4 h. DMSO was used to dissolve the formed formazan, and the absorbance was measured at 570 nm using a microplate reader (Bio-Tek Instruments, USA).

### Cell apoptosis

Cells were seeded into 12-well plates at 1 × 10^5^/well and treated with metformin for 48 h. The cells were washed with PBS 3 times and collected for Annexin/PI analysis according to the manufacturer's instructions. In total, 10000 cells were collected by flow cytometry with a FACSCalibur (BD Biosciences, USA), and the data were analyzed using the Flowjo software.

### Cell cycle analysis

Cells were cultured with metformin for 48 h and then collected. Pre-cooled 70% ethanol was used to permeabilize the cells overnight at 4°C. The cell cycle detection kit was used according to the manufacturer's instructions. Briefly, the cells were washed with PBS, incubated with RnaseA for 30 min at 37°C and PI for 30 min at 4°C. In total, 20000 cells were collected, and the cell cycle distribution was calculated using the Flowjo software.

### Mitochondrial membrane potential

The cells were treated with metformin and collected for JC-1 staining for 20 min at 37°C. In total, 10000 cells were collected by flow cytometry. JC-1 exists either as a green-fluorescent monomer at depolarized membrane potentials (FL1) or as an orange-fluorescent J-aggregate at hyperpolarized membrane potentials (FL2). The median fluorescence intensity (MFI) was analyzed in both FL1 and FL2, and the results were shown as MFI _(FL1)_/MFI _(FL2)_.

### Mitochondrial mass

After metformin treatment for 24 h, the mitochondrial mass was determined by MitoTracker^®^ Green FM (10 nM) and Red CMXRos (20 nM) for 45 min at 37°C. The cells were harvested, and then analyzed by flow cytometry. The results were presented as fold changes in MFI over controls.

### Intracellular ROS production

The cells were treated with metformin for 4 h, and ROS production was evaluated with a Reactive Oxygen Species Assay Kit. The cells were incubated with serum-free 1640 medium diluted DCFH-DA (10 μM) for 20 min at 37°C. Finally, the cells were harvested and MFI_(FL1)_ was recorded.

### Confocal microscopy

For mitochondrial morphology observation, cells were treated with metformin for 48 h and incubated with MitoTracker^®^ Red CMXRos (100 nM) in a serum-free medium for 30 min at 37°C. The cells were washed with PBS and observed under a confocal laser scanning microscope IX81-FV1000 (Olympus, Japan).

For intracellular dox distribution examination, cells were pre-treated with metformin, incubated with doxorubicin (10 μg/ml) for 4 h, followed by nuclei staining with Hochest33342 for 20 min. Then, cells were washed with PBS and observed under CLSM.

### Intracellular ATP production

Cells were inoculated into 96-well plates at a density of 5 × 10^3^/well and treated with metformin. Intracellular ATP was measured using a Celltiter-Glo^®^ Luminescent Kit according to the manufacturer's instructions. The emitted light was associated with ATP concentrations and recorded by a SpectraMax M5 Plate reader (Molecular Devices, USA).

### Quantitative RT-PCR (qRT-PCR)

Cells were inoculated into 6-well plates at a density of 2–3 × 10^5^/well and treated with metformin and doxorubicin for 48 h. Total RNA was extracted by RNAiso Plus, and the concentration or quality was determined by Nanodrop2000 (Thermo Scientific, USA). Primers for qRT-PCR were synthesized by Sangon Biotech Co., Ltd. (Shanghai, China) and are listed in Supplementary Table 1. cDNA was converted from 0.5–1 μg of RNA using PrimeScript™ RT Master Mix, and qRT-PCR was performed with GoTaq^®^ qPCR Master Mix on the LightCycler^®^480 (Roche, Germany). The fold change of gene expression was normalized against the untreated control group using the 2^-ΔΔCt^ method.

### Western blot analysis

The cells were inoculated into 6-well plates at a density of 2–3 × 10^5^/well and treated with drugs for 48 h. Cells were washed with PBS and lysed with RIPA buffer on ice. For tumor tissue, the samples were washed with PBS and homogenated in RIPA buffer on ice (20 mg/200 μl). Equal amounts of proteins (20–30 μg) were separated by 10% NuPAGE^®^ Bis-Tris Pre-Cast Gels and then transferred to 0.45 μm PVDF membranes (Perkin Elmer). The membranes were blocked with 3% non-fat milk, and incubated with primary antibodies overnight at 4°C. HRP-conjugated secondary antibodies were incubated for 2 h at room temperature. The immunoreactive bands were visualized with a BeyoECL Plus kit and ChemiDoc™ Touch Imaging System (Bio-Rad, USA).

### Small interfering RNA transfection

AMPK targeted siRNA and negative control siRNA (siNC) were synthesized by Ribobio Co., Ltd. (Guangzhou, China). The AMPK siRNA sequence was GAGGAGAGCUAUUUGAUUAdTdT. Transfection was performed using a RFect Transfection Reagent with siRNA concentration of 50 nM. Metformin was added 48 h after transfection. Protein was collected 48 h later for western blot analysis.

### MCF7/ADR xenografts nude mice model and *in vivo* anti-tumor study

The *in vivo* anti-tumor activity was accessed in the MCF7/ADR xenografts bearing nude mice. Female mice that were 5 weeks old were subcutaneously injected with MCF7/ADR cells (1 × 10^7^/site) to grow a tumor bulk, which was then cut into small pieces (1 mm^3^) and freshly transplanted into the right flank. When the tumor reached approximately 50 mm^3^, the mice were divided into groups, with 5–6 per group. For metformin treatment, the mice received an intratumoral injection (50 μl) of low-dosage metformin (20 mg/kg) and high-dosage metformin (100 mg/kg) every 3 days. For the combination treatment of doxorubicin and metformin, the mice received an intratumoral injection (total volume of 50 μl) of doxorubicin (0.5 mg/kg) and metformin (100 mg/kg) alone or in combination every 3 days. Physiological saline was used as a control. The tumor volume was monitored using calipers and calculated as V = 1/2 × length × width^2^ (mm^3^). After the treatments, the mice were sacrificed and the tumor bulks were harvested for further studies. All experiments were performed in accordance with the procedures and protocols of the Animal Ethics Committee of Zhejiang University.

### Immunofluorescence staining

The tumor samples were cryosectioned at 8 μm using CM1950 (Leica, Germany). The sections were fixed with ice acetone for 10 min and blocked with goat serum for 1 h. Primary antibodies were incubated with the sections at 4°C overnight, and Alexa Fluor^®^594 conjugated secondary antibodies were incubated for 1 h at room temperature. DAPI was used to label the nuclei. Finally, the sections were photographed with a fluorescence microscope (Eclipse Ni-U; Nikon, Japan).

### Statistical analysis

The results were presented as the mean ± SEM. Statistical analyses were performed with SigmaPolt 10.0 and SPSS 17.0. Differences between groups were evaluated by Student *t* test for comparison of two groups or one-way ANOVA with Tukey's test for more than two groups. *P* < 0.05 was considered statistically significant. The CalcuSyn software version 1.0 was used to calculate the combination index (CI) by the Chou–Talalay method.

## SUPPLEMENTARY MATERIALS FIGURES AND TABLE


