# Supplementary material for: Metformin synergistically suppress tumor growth with doxorubicin and reverse drug resistance by inhibiting the expression and function of P-glycoprotein in MCF7/ADR cells and xenograft models
Source: Oncotarget. 2017 Dec 8;9(2):2158–74. doi: 10.18632/oncotarget.23187 (PMC5788630; doi:10.18632/oncotarget.23187)
Supplement: Supplementary file 1 [file oncotarget-09-2158-s001.pdf]

Metformin synergistically suppress tumor growth with doxorubicin and reverse drug resistance by inhibiting the expression and function of P-glycoprotein in MCF7/ADR cells and xenograft models

SUPPLEMENTARY MATERIALS

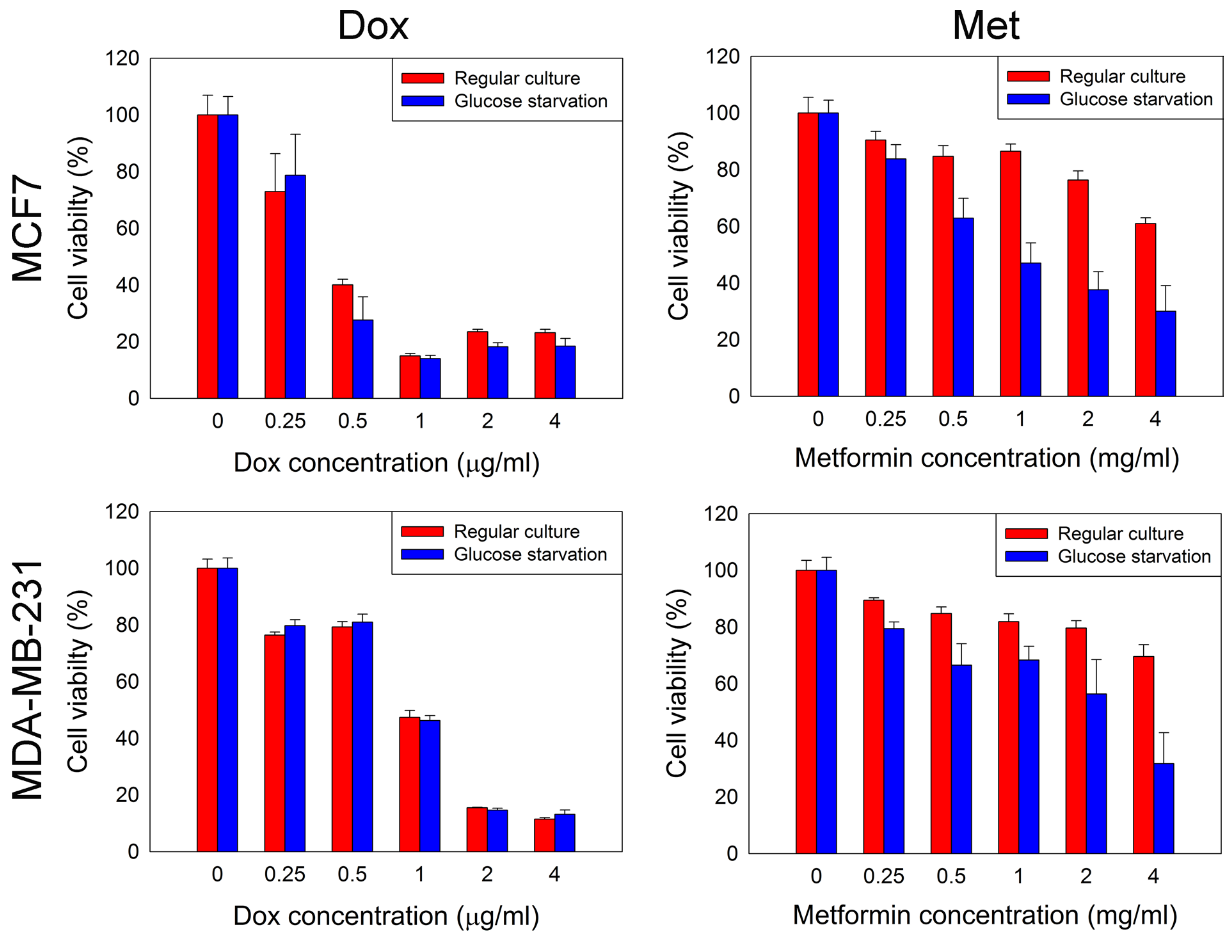

Supplementary Figure 1: Cytotoxicity of metformin and doxorubicin in MCF7 and MB-MD-231 cell line impacted by glucose starvation at 48 h. Cell viability without drug treatment as regarded as 100%, respectively (*n* = 5).

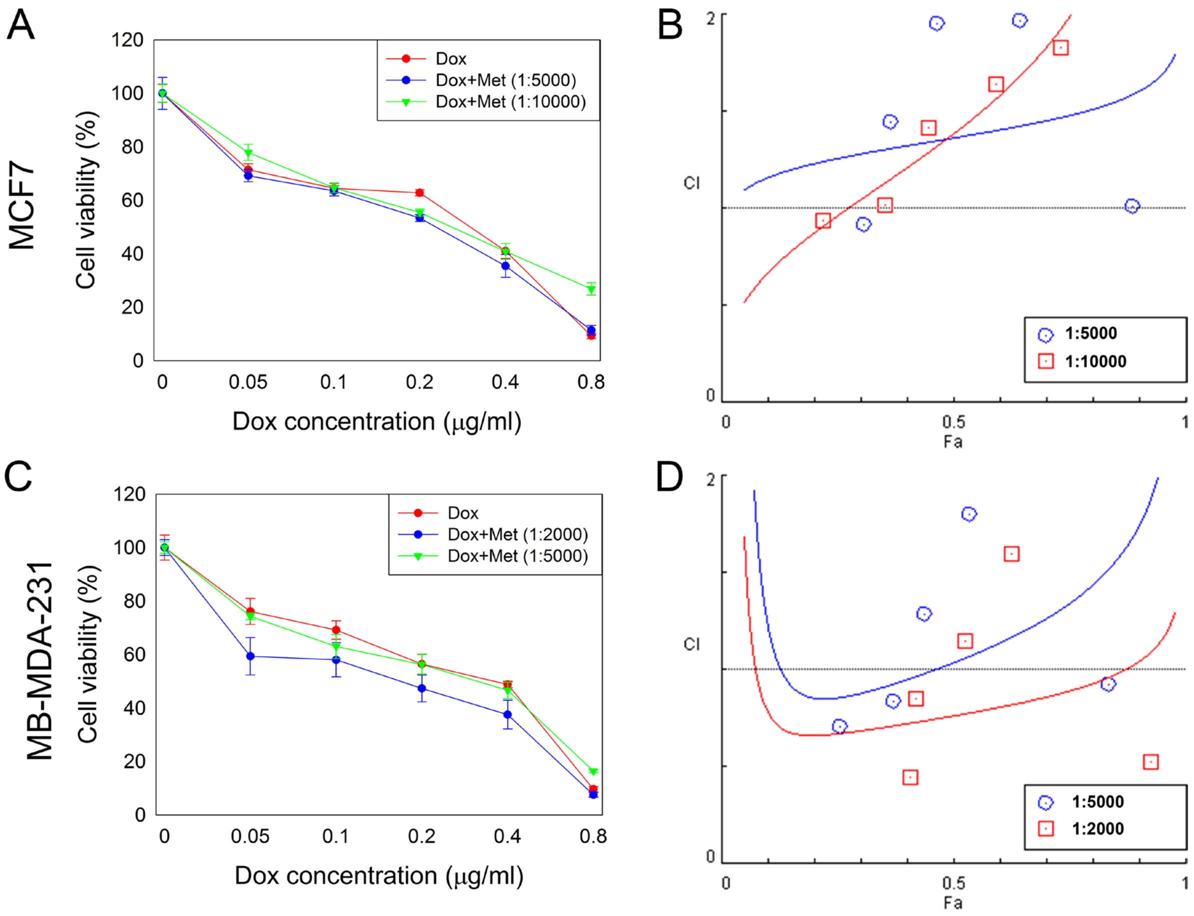

Supplementary Figure 2: Combined effect of metformin with doxorubicin in MCF7 and MB-MDA-231. (**A**, **B**) Effect of metformin and doxorubicin with defined combination ratios on cell proliferation in MCF7 (A), and the corresponding Fa-CI plot of CI (B) (*n* = 5). (**C**, **D**) Effect of metformin and doxorubicin with defined combination ratios on cell proliferation in MB-MDA-231 (C) and the corresponding Fa-CI plot of CI (D) (*n* = 5).

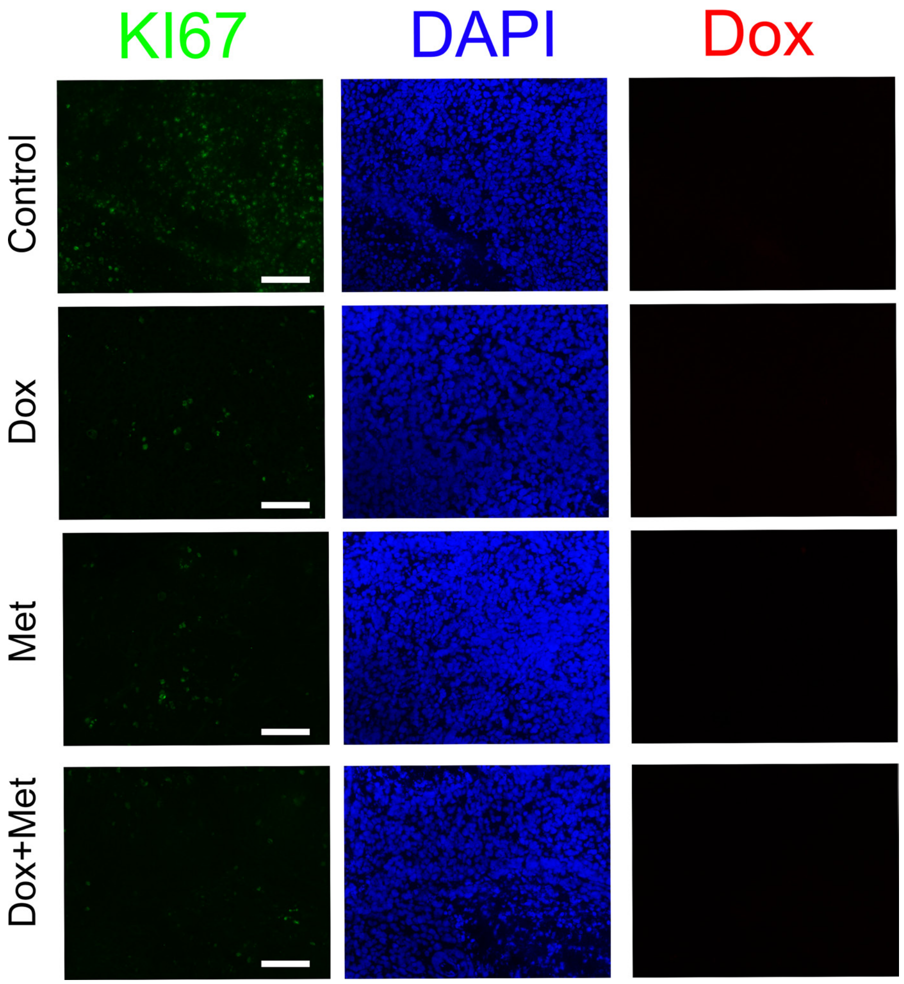

Supplementary Figure 3: Immunofluorescence staining of Ki67 in MCF7/ADR tumor tissues using Alexa Fluor®488 labeled secondary antibodies. Bar = 100 µm.

Supplementary Table 1: Primers for qRT-PCR

Gene	Forward primer	Reverse primer	Product size
ABCB1	TTGCTGCTTACATTCAGGTTTCA	AGCCTATCTCCTGTCGCATTA	105
ABCC1	CTCTATCTCTCCCGACATGACC	AGCAGACGATCCACAGCAAAA	94
ABCC2	TCTCTCGATACTCTGTGGCAC	CTGGAATCCGTAGGAGATGAAGA	115
ABCG2	ACGAACGGATTAACAGGGTCA	CTCCAGACACACCACGGAT	93
Twist1	GGAGTCCGCAGTCTTACGAG	TCTGGAGGACCTGGTAGAGG	200
Snail	TGCGCTACTGCTGCGCGAAT	GGGCTGCTGGAAGGTAAACTCTGGA	147
MMP1	GGGGCTTTGATGTACCCTAGC	TGTCACACGCTTTTGGGGTTT	142
MMP9	CGTCTTCCAGTACCGAGAGA	GCAGGATGTCATAGGTCACG	119
GAPDH	GGAGCGAGATCCCTCCAAAAT	GGCTGTTGTCATACTTCTCATGG	197

Abbreviations: ABCB1, ATP Binding Cassette Subfamily B Member 1; ABCC1, ATP Binding Cassette Subfamily C Member 1; ABCC2, ATP Binding Cassette Subfamily C Member 2; ABCG2, ATP Binding Cassette Subfamily G Member 2; MMP1, Matrix Metallo peptidase 1; MMP9, Matrix Metallo peptidase 9; GAPDH, Glyceraldehyde-3-Phosphate Dehydrogenase.
